# Restoring Oral Health: Implant-Supported Full-Mouth Rehabilitation for an Edentulous Patient

**DOI:** 10.7759/cureus.66732

**Published:** 2024-08-12

**Authors:** Lisa Debbarma

**Affiliations:** 1 Prosthodontics, Rajasthan University of Health Sciences College of Dental Sciences, Jaipur, IND

**Keywords:** implant-supported fixed prosthesis, open tray impressions, pattern resin, hybrid dentures, edentulous patients

## Abstract

Edentulism, the loss of all the natural teeth, significantly impacts a patient's functional, aesthetic, and psychological well-being. Traditional dentures often fail to provide the required stability and functionality. Implant-supported full-mouth rehabilitation has emerged as an advanced solution, leveraging strategically placed dental implants to support fixed prostheses that mimic natural teeth in appearance and function. This case report details the comprehensive treatment of a 78-year-old male patient with complete edentulism, utilizing six implants in both the maxillary and mandibular arches. Following a five-month healing period, a series of precise prosthetic procedures, including abutment-level impressions, custom tray fabrication, and implant-level impressions, were performed to ensure optimal fit and functionality. The final prostheses provided significant improvements in masticatory efficiency, speech, and overall quality of life. The report underscores the transformative potential of implant-supported rehabilitation, highlighting high success rates, patient satisfaction, and the multifaceted benefits of restoring oral function and aesthetics with advanced dental technologies.

## Introduction

The loss of all the natural teeth, known as edentulism, poses a significant clinical challenge that impacts both the functional and aesthetic aspects of a patient's life. Edentulism not only affects the ability to chew and speak but also diminishes the overall quality of life, leading to psychological and social consequences. Traditional solutions like complete dentures, while common, often fall short of providing the stability and functionality required for optimal oral health [1]. Implant-supported full-mouth rehabilitation has emerged as a revolutionary approach to address these shortcomings. This treatment modality involves the strategic placement of dental implants to support fixed prostheses, offering a stable and permanent solution that mimics natural teeth in function and appearance. The use of dental implants for full mouth rehabilitation not only restores masticatory efficiency and speech but also significantly enhances the patient's confidence and overall well-being [2].

Recent advancements in implant technology and surgical techniques have further refined this approach, allowing for immediate loading and the use of computer-aided design and manufacturing (CAD/CAM) to create precise and aesthetically pleasing prosthetic solutions. Studies have demonstrated high success rates and patient satisfaction with implant-supported rehabilitations, underscoring their efficacy as a long-term solution for edentulous patients. For instance, a multicenter retrospective analysis reported a 97.9% implant survival rate and significant patient satisfaction improvements with fixed prostheses using computer-aided flapless implant placement compared to conventional dentures [3]. Immediate loading of both upright and tilted implants has also shown comparable outcomes, achieving high patient satisfaction in terms of aesthetics, phonetics, and function [4].

In this case report, we explore the application of implant-supported full-mouth rehabilitation in a completely edentulous patient, highlighting the procedural steps, clinical outcomes, and significant improvements in the patient's quality of life. Through comprehensive diagnosis, meticulous planning, and advanced surgical techniques, we aim to demonstrate the transformative potential of this treatment modality.

## Case presentation

A 78-year-old male reported at Government Dental College and Hospital, Jaipur, India, with the chief complaint of missing teeth in the upper and lower jaws. The patient was then referred to the Department of Oral and Maxillofacial Surgery. After a thorough investigation and treatment planning, six implants in the maxillary arch and six implants in the mandibular arch were placed. After a healing period of five months, the patient was referred to the Department of Prosthodontics and Crown and Bridges.

Prosthetic phase

**Figure 1:**
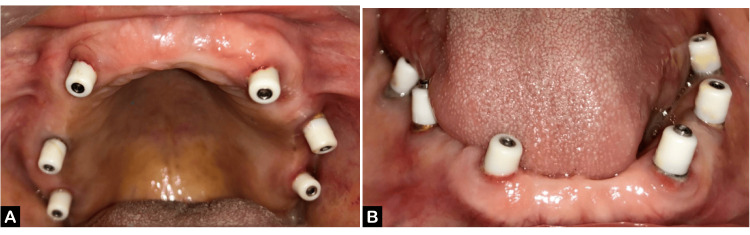
Healing abutments in the patient's mouth (A) Maxillary arch and (B) mandibular arch.

The patient reported at the Department of Prosthodontics with 12 healing abutments placed in the maxillary and mandibular arches (Figure 1A, 1B).

**Figure 2:**
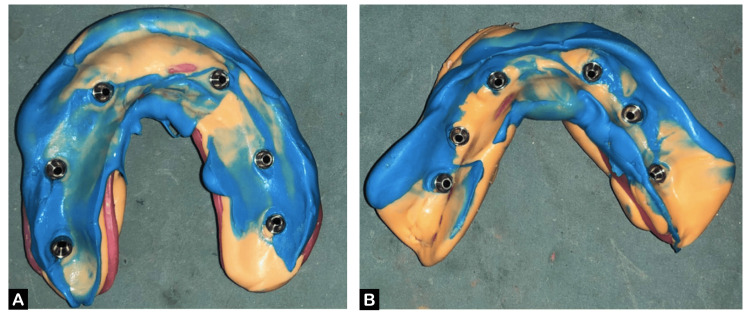
Master impression (A) Maxillary arch and (B) mandibular arch.

An abutment-level impression was planned with an alginate impression material (WaldentFlexiPrint Alginate, WaldentAlChem, New Delhi, India) for the purpose of making primary casts (maxillary and mandibular). The primary casts were used to fabricate custom trays; a 1 mm thick modeling wax spacer was adapted over the cast with window cut out over implant areas, and trays were fabricated by using a self-cure acrylic resin (DPI RR, Mumbai, India) for open tray impression. An open tray, implant-level impression (master impression) for both the maxillary and mandibular arches was planned. During the procedure, the healing abutments were removed and replaced with transfer copings, which were then securely tightened into each fixture. These transfer copings were splinted with the help of pattern resin. Custom trays were tried intraorally, loaded with polyvinyl siloxane elastomeric impression material (GC Flexceed; GC India, Telangana, India), and placed in the mouth. The tray was held in position until it was set, followed by unscrewing the transfer coping. Then the impression was removed from the mouth. The same procedure was followed for the opposing arch (Figure 2A, 2B).

**Figure 3:**
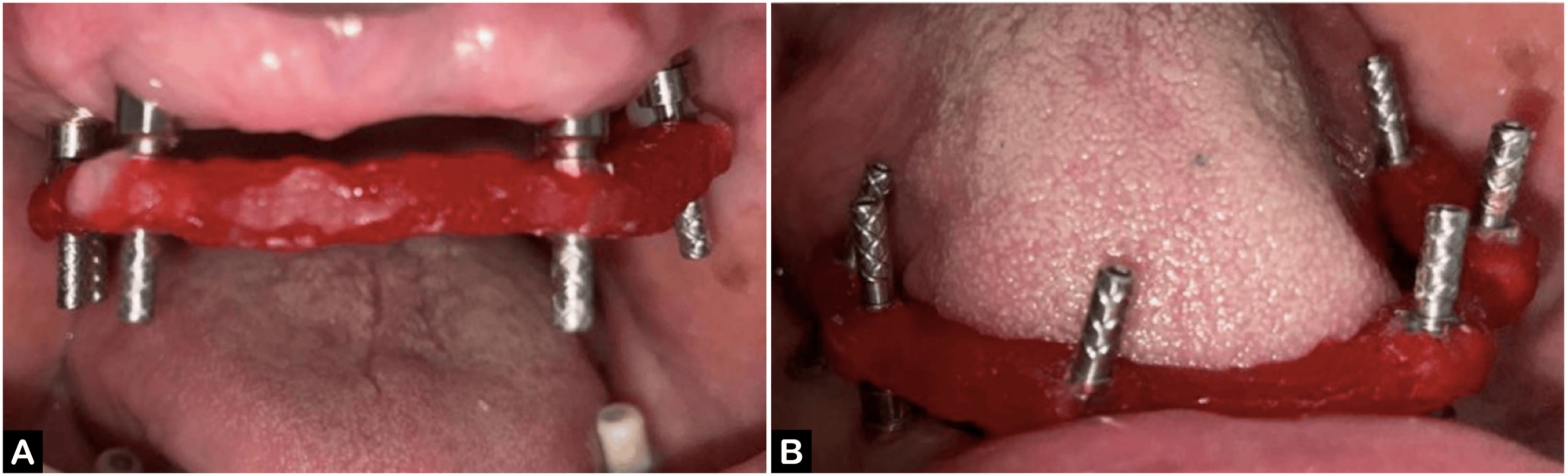
Jig trial verification (A) Maxillary arch and (B) mandibular arch.

**Figure 4:**
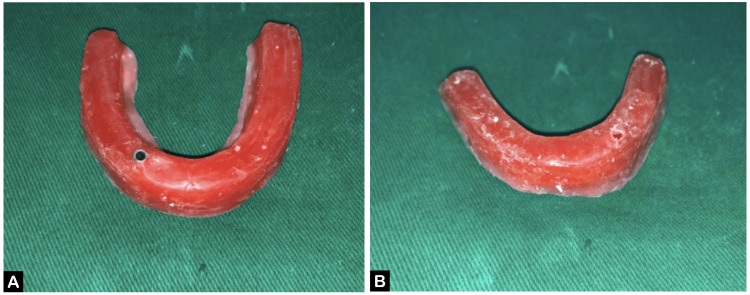
Occlusal rims (A) Maxillary arch and (B) mandibular arch.

Denture bases were fabricated. A resin jig was created using pattern resin (GC America Inc., Alsip, Illinois, United States) on the cast, with definitive abutments in place, verified for any discrepancies (Figure 3A, 3B). Occlusal rims were created for recording the jaw relation (Figure 4A, 4B).

**Figure 5:**
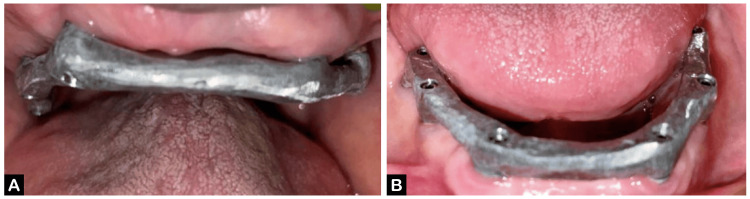
Metal framework trial (A) Maxillary arch and (B) mandibular arch.

**Figure 6:**
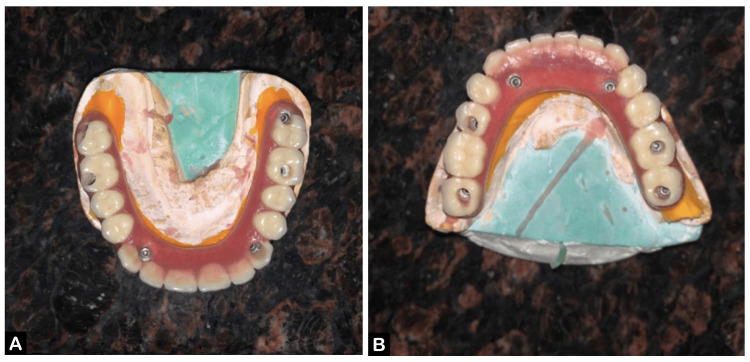
Hybrid denture (A) Maxillary arch and (B) mandibular arch.

After recording the jaw relations, the casts were mounted on a semi-adjustable articulator, and the teeth arrangement was performed. A try-in was conducted to check occlusion, fullness, and visibility, followed by the fabrication of a metal framework. The framework was tried in the mouth, and shade selection was completed (Figure 5A, 5B). The maxillary and mandibular hybrid prosthesis were fabricated (Figure 6A, 6B).

**Figure 7:**
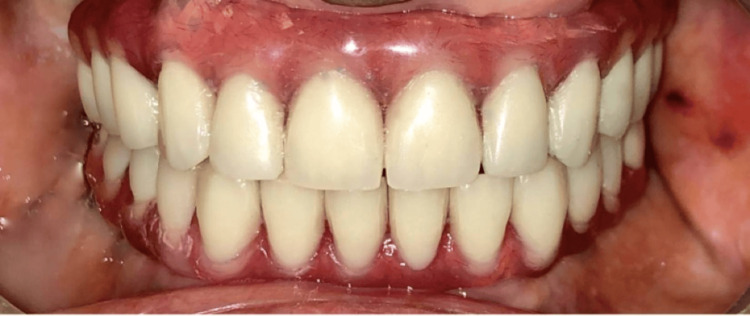
Hybrid dentures in the patient’s mouth Multiunit abutments were torqued to 15 Ncm, and the screw access holes were sealed with Teflon tape and flowable composite.

**Figure 8:**
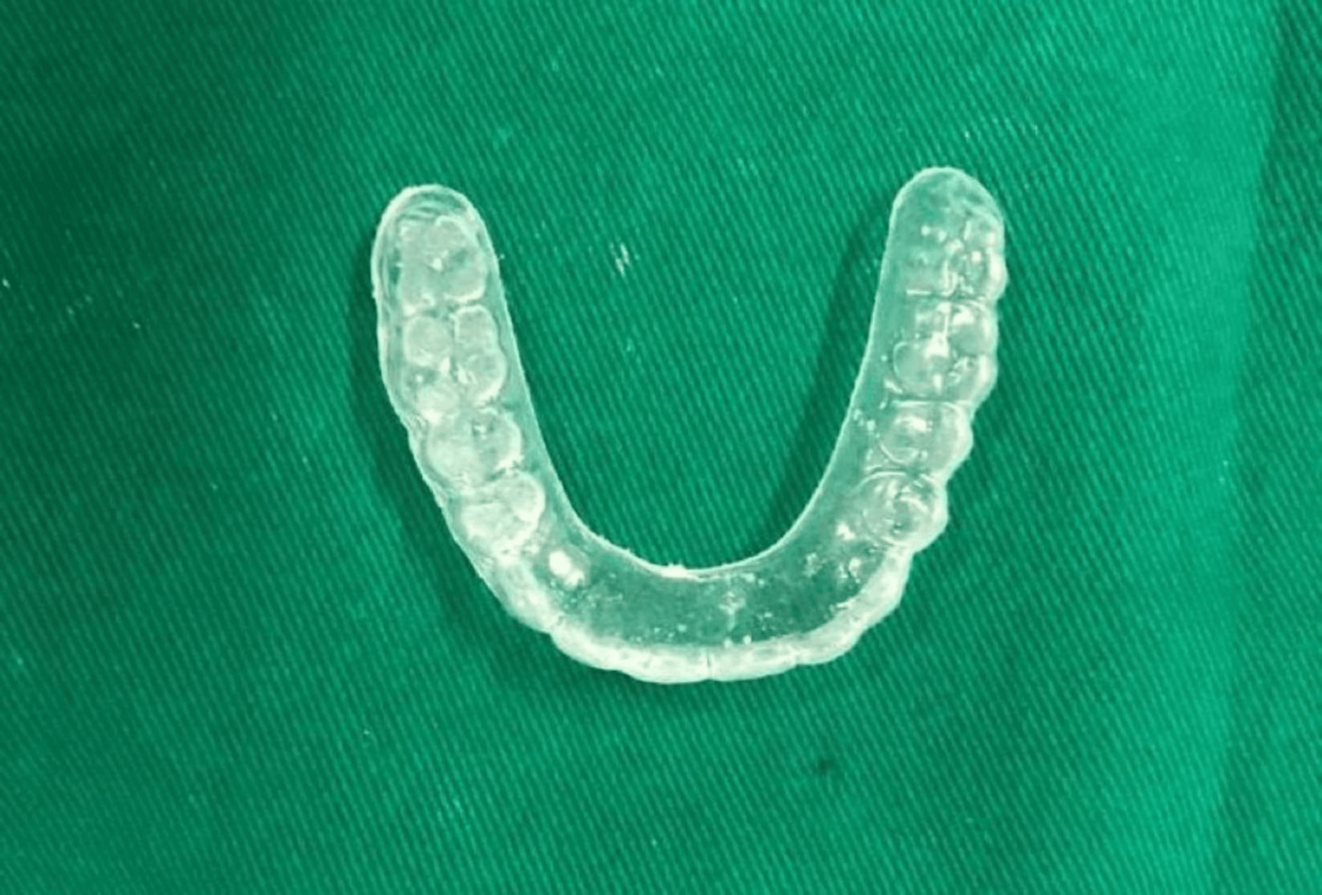
Night guard Maxillary night guard was provided to the patient.

The multiunit abutments were torqued to 15 Ncm, the screw access was blocked with Teflon tape and sealed with a flowable composite, and a minor occlusal correction was done with the help of an articulating paper (Figure 7). A night guard was provided (Figure 8). The patient was then recalled after 24 hours and a month.

## Discussion

Implant-supported full-mouth rehabilitation represents a pioneering advancement in modern dental care, offering a comprehensive solution for individuals who have lost most or all of their natural teeth due to various reasons such as decay, trauma, or periodontal disease. This modern technique includes the surgical placement of biocompatible titanium dental implants into the jawbone, where they act as strong anchors for fastidiously created prosthetic teeth. These implants mimic the function of natural tooth roots, providing a stable foundation that supports the prosthetics with remarkable durability and reliability [5].

The multifaceted benefits of implant-supported full-mouth rehabilitation are well documented in the dental literature. Functionally, these prosthetics restore the ability to chew a wide variety of foods comfortably and speak articulately, offering significantly improved performance compared to conventional removable dentures. The stability provided by implants helps distribute chewing forces evenly across the jawbone, which not only enhances chewing efficiency but also mitigates the risk of bone resorption and subsequent facial collapse over time [6].

Aesthetic considerations are equally profound, as the prosthetic teeth are custom-designed to harmonize seamlessly with any remaining natural dentition and complement the patient's facial features. Restoring a natural smile not only enhances self-esteem and overall confidence but also improves facial aesthetics by maintaining proper lip support and contour. However, achieving implant-supported full-mouth rehabilitation comes with its challenges [7-10].

The procedure requires a sufficient amount of healthy jawbone to support the implants securely. Patients with inadequate bone volume or density may need preliminary procedures such as bone grafting or sinus lifts to augment the jawbone and create a suitable foundation for implant placement [11-14]. Moreover, the treatment process typically spans several months and demands meticulous planning by a collaborative team of dental specialists including prosthodontists, oral surgeons, and dental technicians.

While the initial costs associated with implant-supported full-mouth rehabilitation may be higher compared to traditional dentures, many patients view it as a worthwhile long-term investment in their oral health and quality of life. Dental implants are renowned for their exceptional durability and longevity, often lasting a lifetime with proper care and maintenance. This durability, coupled with the restoration of natural chewing function and aesthetic appeal, significantly enhances overall well-being and satisfaction for patients seeking a permanent solution to extensive tooth loss [15].

## Conclusions

Implant-supported full-mouth rehabilitation is a transformative treatment for edentulous patients, significantly enhancing both function and aesthetics. Dental implants provide a stable, durable foundation for prosthetic restorations, offering clear advantages over traditional dentures.

High success rates and patient satisfaction are achieved through effective osseointegration and advanced surgical techniques, minimizing complications. This approach improves masticatory efficiency, allowing for a more varied diet and better overall health. Additionally, the psychological benefits of a fixed, natural-looking set of teeth boost self-esteem and social interactions.
